# The effect on turnout of campaign mobilization messages addressing ballot secrecy concerns: A replication experiment

**DOI:** 10.1371/journal.pone.0182199

**Published:** 2017-08-09

**Authors:** Alan S. Gerber, Gregory A. Huber, Albert H. Fang, Catlan E. Reardon

**Affiliations:** 1 Department of Political Science, Yale University, New Haven, Connecticut, United States of America; 2 Institution for Social and Policy Studies, Yale University, New Haven, Connecticut, United States of America; 3 Travers Department of Political Science, University of California at Berkeley, Berkeley, California, United States of America; University of Vermont, UNITED STATES

## Abstract

Given the persistence of public doubts about the integrity of ballot secrecy, which depress turnout, two prior experiments have shown precise evidence that both official governmental and unofficial mobilization campaigns providing assurances about ballot secrecy increase turnout among recently registered nonvoters. To assess whether these findings replicate in other political settings, we describe a replication experiment where a non-governmental, non-partisan mobilization campaign sent similar treatment mailings containing assurances about ballot secrecy protections to recently registered nonvoters during the 2014 general election in Mississippi. We find that sending this mailer has no effect on turnout rates in this setting, which is characterized by an unusually low baseline turnout rate. These results are consistent with past research concluding that nonpartisan Get Out The Vote (GOTV) mail has very weak effects among very low turnout propensity registrants, and suggest that there are heterogeneous effects of ballot secrecy treatments associated with subjects’ characteristics and the electoral context.

## Introduction

Doubts about the integrity of ballot secrecy protections are prevalent among nonvoters [1, 2] and have been linked to depressed levels of political participation [2, 3]. Accordingly, there has been considerable interest among practitioners and scholars alike in whether Get Out the Vote (GOTV) appeals that provide assurances about ballot secrecy protections are effective at increasing turnout levels among registered nonvoters.

To date, there are two field experiments that provide precise evidence that such treatments, which encourage individuals to vote and communicate assurances about ballot secrecy protections, are effective at increasing turnout among recently registered nonvoters regardless of whether the appeal is sent by an official governmental source [2] or by a non-governmental and non-partisan source [4]. The first of these studies reports findings from a field experiment conducted in Connecticut in 2010, in which sending a letter from the Secretary of State providing information about ballot secrecy protections increases participation levels among registered nonvoters by about 3.8 percentage points (p<.01, two-tailed; n = 69,488) when compared to registered nonvoters in a placebo condition that is sent, from the same source, election-related mail that does not address ballot secrecy concerns [2]. The second study examines whether nearly identical ballot secrecy treatments are effective at increasing turnout levels when sent from a non-governmental source [4]. Analyzing data from a large field experiment targeting recently registered nonvoters in six states (Arkansas, Georgia, Louisiana, Michigan, North Carolina, and Texas). in the 2014 general election, the authors of that study report that sending the ballot secrecy treatment mailing increases turnout levels by about 1 percentage point among registered nonvoters (p<.01, two-tailed; n = 281,929) when compared to a control group that was sent no mailing [4]. (In addition, an earlier study by [5] also offers suggestive evidence of positive effects when the appeal is sent from a non-governmental source, but this study was statistically underpowered to detect small effects.)

To assess whether the treatment effects reported in these previous experiments are reproducible in other political settings, this article reports findings from a replication field experiment in which a non-governmental and non-partisan mobilization campaign randomly sent GOTV appeals addressing ballot secrecy concerns (i.e., mailers containing a bundled treatment, which is virtually identical to that tested in previous experiments) to recently registered nonvoters in Mississippi during the 2014 general election. The treatment we test is a single mailer that communicates three main appeals: a standard GOTV mobilization appeal, information about potential threats to ballot secrecy, and information about the integrity of electoral institutions and practices that is designed to directly ameliorate these concerns. We find no effect of sending the ballot secrecy treatment mailing on turnout levels when compared to a control group that was sent no mailing. The remainder of this essay describes the experimental design, presents results, and discusses the implications of the findings, in particular the need for future research to isolate the effects of the specific appeals relating to ballot secrecy contained in the bundled treatment and to assess whether different subjects are responding to different parts of the bundled treatment.

## Experimental design

We designed and analyze data from a replication field experiment conducted in Mississippi during the 2014 general election. This was a low salience election, as there were no competitive statewide or congressional races and no controversial initiatives on the ballot in the 2014 general election in Mississippi. In the experiment, a consulting firm specializing in direct voter contact programs sent GOTV appeals addressing ballot secrecy concerns to a randomly selected group of recently registered nonvoters.

The experiment proceeded as follows. First, the firm obtained a list of eligible Mississippi registrants from an outside private vendor that regularly collects and standardizes voter file records from the state and merges that data with vote history and consumer file records. The private vendor processed that file and verified registrants’ addresses using a National Change of Address filter.

Second, the firm selected the study population of recently registered nonvoters from this list of registrants. Consistent with existing experiments in the literature [2, 5], recently registered nonvoters are defined as individuals who have never voted in any prior election, who had registered to vote since the general election 6 years prior, and had not ever voted, including not voting in at least one high-salience presidential election. Applying this definition to this study, we operationalize subjects as individuals who reside in and are registered to vote in Mississippi, who registered to vote between November 5, 2008 (the day after the 2008 general election) and before October 1, 2012, and who never voted in any prior elections. Subjects are randomly selected from households such that no two subjects are from the same household. This yields 12,738 subjects in the experiment, who are predominantly black (77.6%), roughly balanced by gender (about 43% female, 42.5% male, and 14.6% of unknown gender), and have a high predicted likelihood of being Democratic (mean = 84.6%, s.d. = 10.5%). (Note: The Democratic likelihood score is a proprietary measure provided by the private vendor and is modeled as a function of pre-treatment subject-level demographic characteristics.)

Subjects were randomly assigned to a treatment group (n = 8,704) that was sent a GOTV appeal addressing ballot secrecy concerns, which we describe in greater detail below, or to a control group (n = 4,034) that was sent no mailing. The randomization procedure occurred at the subject-level. A randomization check regressing treatment assignment on observed covariates confirms that the covariates are not jointly prognostic of treatment (F = .8, p = .62) and we infer that the randomization procedure is valid. Details about the randomization check and a balance table are presented in S1 Appendix.

The ballot secrecy treatment mailer was sent five days before the election by the Mississippi Center for Voter Information, a nonpartisan nonprofit organization. The treatment mailing is a letter that begins by reminding subjects about when Election Day is and when the polls are open. The letter continues by providing assurances about ballot secrecy protections that are phrased identically to those tested in prior experiments. Specifically, these assurances are designed to mitigate three types of fears citizens might have about ballot secrecy. First, the treatment letter assures subjects that their vote choices cannot be traced back to their name. Second, the treatment letter assures subjects that voting booths are private places to vote. Third, the letter assures subjects that voting is free of intimidation from polling workers or campaigns. In order to construct a coherent appeal, the treatment script in this and previous experiments raises potential concerns about ballot secrecy before addressing them. The treatment mailing concludes by directing subjects to the Mississippi Secretary of State for more information about the voting process and urging the subject to vote and participate in the democratic process. A sample mailing containing the full treatment text is provided in S1 Appendix.

Following the election, we obtained participation records for all subjects from the same vendor. The outcome variable of interest is turnout in the 2014 general election, which is coded 1 if the subject voted and 0 otherwise.

## Results

Using ordinary least squares with inverse probability weights, we regress turnout in 2014 on treatment assignment and a battery of observed demographic covariates, which include age, gender indicators, race and ethnicity indicators, the number of days between Election Day and their voter registration date, and indicators to denote if the value of age or days since registering to vote are missing. We impute missing values for all variables using the sample mean of that variable and include as a covariate a binary indicator variable that equals 1 if the covariate value is imputed. Weights are defined as the inverse of the probability of assignment to the observed treatment assignment.

Results are shown in Table 1. We focus in particular on the first column, which presents the estimates from our primary and preferred specification. The baseline mean turnout rate in the control group is unusually low at 1.4 percent. When compared to the control group, sending the ballot secrecy treatment has a negative but virtually null effect on turnout that is statistically indistinguishable from zero (estimate = −0.0005; s.e. = 0.0020; *p* = 0.828, two-tailed). The remaining columns show that this main finding is not materially affected by the use of covariate adjustment or inverse probability weights.

**Table 1:** Sending the ballot secrecy treatment mailing has no effect on turnout in the 2014 election.

Variable	(1) Weighted and With Covariates	(2) Weighted and Without Covariates	(3) Unweighted and With Covariates	(4) Unweighted and Without Covariates
Ballot Secrecy Treatment (1 = Yes)	-0.00049	-0.00036	-0.00050	-0.00036
	(0.00224)	(0.00224)	(0.00224)	(0.00224)
Constant	0.03261	0.01425	0.03237	0.01425
	(0.00764)	(0.00127)	(0.00770)	(0.00127)
Observations	12,738	12,738	12,738	12,738
Weighted?	Yes	Yes	No	No
With Covariates?	Yes	No	Yes	No
Control Group Mean Turnout	0.0142	0.0142	0.0142	0.0142

Cells report coefficient estimates from an ordinary least squares regression, with robust standard errors in parentheses. None of the treatment effect estimates reported in the table are statistically significant at the 0.01 level. The dependent variable is turnout in the 2014 general election, coded 1 if the subject voted and 0 otherwise. The omitted comparison group is the control group. Estimates on covariates are not shown in this table. We refer the reader to S1 Appendix for a full set of regression estimates.

## Discussion

This null result contrasts against the positive effects of ballot secrecy treatments reported in previous large-scale experiments. We speculate ex post that this null result may be due to heterogeneous treatment effects associated with characteristics of the sample and/or electoral context, and we analyze ancillary data to assess the plausibility of these claims.

First, we assess the similarity of the control group in this study to comparable control groups in experimental studies fielded at the same time. Specifically, we compare the baseline turnout rate in the control group in this study (in Mississippi) to state-specific control group turnout rates in [4], which also tests the effect of non-governmental ballot secrecy GOTV appeals on recently registered nonvoters (which are defined similarly) in states other than Mississippi in the same general election in 2014. Whereas the state-specific baseline turnout rates in the experiment by [4] range from 10% to 23%, the control group turnout rate in this study unusually low at 1.4%. This is unusually low even when compared to Mississippi’s low statewide turnout rate of 20.8% in 2014. (Note: Using data from the Mississippi Secretary of State, the statewide turnout rate in 2014 is calculated by dividing the total number of ballots cast for the highest office with an election—630,858 for the U.S. Senate election—by the total number of registered voters in the state—3,040,740.) This suggests that even among the pool of recently registered nonvoters, the Mississippi sample had unusually low turnout. In light of prior experimental findings by [2] that GOTV appeals addressing ballot secrecy concerns are more effective than standard GOTV appeals, the findings from this study also suggest that for the sample of recently registered nonvoters in Mississippi examined in this study, standard GOTV appeals would also be ineffective at increasing turnout. More generally, the null result is consistent with findings in the field experimental literature on voter mobilization that nonpartisan GOTV mail has weak or no effects among voters with extremely low propensities to vote [6].

Second, we assess differences in state-specific political contexts during the 2014 general election to explore possible contextual factors that may explain why subjects’ untreated and treated average turnout rates appear virtually identical during the 2014 general election in Mississippi and appear to be different across the six states in [4]. One possible explanation is that subjects may be more difficult to mobilize if treated during elections with no competitive or controversial contests. This would be true if subjects can be mobilized only if they believe they have an instrumental or normative reason to vote (e.g., they perceive that there is a social choice that they should be weighing in on, either to affect the outcome or to fulfill their role as citizens). In 2014, there were no competitive statewide or congressional races and no controversial initiatives on the ballot in Mississippi, whereas in each of the six studies examined in the experiment by [4] there was either a contested Senate or gubernatorial election or at least one ballot initiative that was potentially controversial.

An important limitation of this and previous experiments is that they test bundled secrecy-related appeals. While a previous placebo-controlled experiment by [2] suggests that secrecy-related appeals sent by an official governmental source are effective at increasing turnout above and beyond a standard GOTV mobilization appeal by approximately 4 percentage points among the target population of recently registered nonvoters, this and previous experiments are not able to isolate the effect of the appeal of the ballot secrecy assurances from the effect of information communicating that potential threats to ballot secrecy exist (i.e., the reason why ballot secrecy assurances are being communicated). For example, it is not possible to rule out that some treated subjects in our sample are less likely to vote because the portion of the treatment letter reminding them that voter intimidation is illegal is inadvertently priming them to think that they might face intimidation at the polls and causing them to not vote in order to avoid the prospect of intimidation. We speculate, however, that expectations that the treatment mailer depresses turnout by heightening expectations of voter intimidation are unlikely for two reasons. First, when read in context, the language in the treatment mailer reminding subjects that voter intimidation is illegal arguably strengthens the credibility and coherence of the ballot secrecy protection assurances communicated in the treatment letter. Second, the electoral context in which the experiment was conducted (a low-salience, low-turnout, non-competitive election) was one in which voter intimidation was not likely to occur (as compared to a highly competitive or salient election where a candidate or party is expected to have an incentive to engage in voter intimidation). Moreoever, the fact that prior studies by [2, 4] found *positive* effects of the bundled treatment (containing identical language reminding subjects that voting is free of intimidation of any kind) on turnout in higher-salience electoral contexts suggests that (i) if there are subjects who avoid voting as a result of the mailer, then the share of voters who would be intimidated by the treatment mailer is substantially smaller than the share of voters who would not be intimidated by the mailer (otherwise these prior studies would have found null or negative effects), and (ii) the effect of ballot secrecy assurances on turnout among subjects who are not intimidated from voting due to the treatment mailer would be larger than the estimates reported in previous experiments. Nevertheless, there remains a general need for future experimental research to better understand whether bundled secrecy-related appeals have heterogeneous effects across different subject pools—either because people are responding to different parts of the combined appeal or because different types of people are responding differently to the same part of a combined appeal—and across different electoral settings.

Taken together, the results suggest four important avenues for future research. First, additional experimental replication is needed to amass a sampling distribution of the effects of mobilization campaigns addressing ballot secrecy concerns on turnout and to understand the generalizability of existing findings. Second, additional research is needed to assess whether the effects of such mobilization appeals depend on specific features of subjects’ electoral and political context that might affect the likelihood a recently registered nonvoter becomes a marginal voter if they are treated. For example, it may be the case that recently registered nonvoters do not become marginal voters if treated in electoral settings where citizens lack both instrumental or expressive reasons to vote. Alternatively, in contexts where citizens’ levels of trust toward governmental and civil society institutions are low at baseline, null effects may be observed because this low level of trust causes citizens to have an extremely low propensity to vote at baseline *and* to be unreceptive to mobilization appeals that attempt to boost confidence in the integrity of electoral institutions. Third, future experimental designs should test the effect of specific appeals contained in the bundled treatment examined in this and in previous experiments. Fourth and finally, future experiments should also be designed to investigate potential heterogeneity in whether subjects are responding to different appeals in these bundled treatments.

## Supporting information

S1 Appendix Supplementary materials. This document contains additional information about the treatment design and empirical analyses.
(PDF)
