# Supplementary material for: The effect on turnout of campaign mobilization messages addressing ballot secrecy concerns: A replication experiment
Source: PLoS One. 2017 Aug 9;12(8):e0182199. doi: 10.1371/journal.pone.0182199 (PMC5549925; doi:10.1371/journal.pone.0182199)
Supplement: S1 Appendix — This document contains additional information about the treatment design and empirical analyses. (PDF) [file pone.0182199.s001.pdf]

Supplemental Appendix for:

THE EFFECT ON TURNOUT OF CAMPAIGN MOBILIZATION MESSAGES ADDRESSING BALLOT SECRECY CONCERNS: A REPLICATION EXPERIMENT

FOR ONLINE PUBLICATION ONLY

This appendix contains the following material:

A: Sample Treatment Mailing

B: Additional Tables

C: Sample Size Calculation

A Sample Treatment Mailing

Figure A1: Sample Ballot Secrecy Treatment Mailing

[redacted]
*************AUTO**3-DIGIT 397 18-1-5501
Tyrone [redacted]
[redacted]
Columbus MS [redacted]

Dear Tyrone,

I want to remind you that the election will be held on Tuesday, November 4th. Polls will be open from 7 am to 7 pm on Election Day.

Your ballot is secret. Poll workers keep only a list of who voted, not how they voted. No record of how you or any other voter filled out their ballot is created. Your ballot choices cannot be matched up with your name.

Additionally, voting booths provide a private place for you to fill out your ballot. You mark your ballot without anyone else looking at it.

Voting is free of intimidation of any kind. A set of rules is enforced at each polling place to ensure that voters are comfortable casting votes for whomever they prefer. For example, poll workers are not permitted to ask you for whom you voted, and campaigning is prohibited inside of or within 150 feet of any entrance to a polling place.

In Mississippi, elections are administered by the Mississippi Secretary of State. If you have any questions about the voting process, please visit their website at www.sos.ms.gov. You can also call 800-829-6786 with any questions you have.

No matter who you are planning to support, we hope you vote and participate in the democratic process!

Sincerely,

Alfred Johnson

Alfred Johnson, President
Mississippi Center for Voter Information

B Additional Tables

Table A1: Randomization Check. This table presents OLS estimates from a regression of treatment assignment on observed covariates, with and without inverse probability weights. At the bottom of the table reports the F-statistic and p-value from a test of the null hypothesis that all the coefficients are equal to zero. We fail to reject the null hypothesis that the observed covariates are jointly prognostic of treatment assignment and infer that the randomization procedure did not fail ($F = .80, p = .62$ for the weighted model).

	(1)	(2)
Variables	Weighted	Unweighted
Age	-0.000	-0.000
	(0.000)	(0.000)
Gender: Female (1=Yes)	-0.004	-0.003
	(0.010)	(0.009)
Gender: Unknown (1=Yes)	-0.022	-0.019
	(0.014)	(0.013)
Race/Ethnicity: Black (1=Yes)	0.006	0.005
	(0.011)	(0.010)
Race/Ethnicity: Hispanic (1=Yes)	-0.006	-0.005
	(0.047)	(0.044)
Race/Ethnicity: Other (1=Yes)	0.007	0.006
	(0.048)	(0.045)
Days Since Registering to Vote	-0.000	-0.000
	(0.000)	(0.000)
Missing Age (1=Yes)	-0.006	-0.005
	(0.009)	(0.009)
Missing Days Since Registering to Vote (1=Yes)	-0.501	-0.317
	(0.414)	(0.329)
Constant	0.543***	0.354***
	(0.030)	(0.027)
Observations	12,738	12,738
R-squared	0.001	0.000
F-statistic	0.800	0.650
F-stat p-value	0.620	0.760

Standard errors in parentheses
*** p<0.01, ** p<0.05, * p<0.1

Table A2: Balance Table.

Variable	Control	Treatment
Age	40.2793	40.1359
	[10.2628]	[10.2495]
Gender: Female (1=Yes)	0.429	0.4308
	[.495]	[.4953]
Gender: Unknown (1=Yes)	0.1484	0.1393
	[.3556]	[.3463]
Race/Ethnicity: Black (1=Yes)	0.7748	0.7786
	[.4177]	[.4152]
Race/Ethnicity: Hispanic (1=Yes)	0.0093	0.0089
	[.096]	[.0941]
Race/Ethnicity: Other (1=Yes)	0.0088	0.0089
	[.0936]	[.0941]
Days Since Registering to Vote	1448.498	1440.843
	[326.6446]	[317.7326]
Missing Age (1=Yes)	0.6374	0.6301
	[.4808]	[.4828]
Missing Days Since Registering to Vote (1=Yes)	0.0002	0
	[.0152]	[0]
Observations	8704	4034

Cells present weighted means and weighted standard deviations in brackets.

Table A3: Sending the Ballot Secrecy Treatment Mailing Has No Effect on Turnout in the 2014 Election. This table presents the full set of regression estimates.

Variable	(1) Weighted and With Covariates	(2) Weighted and Without Covariates	(3) Unweighted and With Covariates	(4) Unweighted and Without Covariates
Ballot Secrecy Treatment (1=Yes)	-0.00049	-0.00036	-0.00050	-0.00036
	(0.00224)	(0.00224)	(0.00224)	(0.00224)
Age	-0.00010		-0.00008	
	(0.00012)		(0.00012)	
Gender: Female (1=Yes)	0.00249		0.00077	
	(0.00243)		(0.00230)	
Gender: Unknown (1=Yes)	0.00043		-0.00148	
	(0.00344)		(0.00306)	
Race/Ethnicity: Black (1=Yes)	-0.00094		-0.00132	
	(0.00281)		(0.00270)	
Race/Ethnicity: Hispanic (1=Yes)	-0.01401***		-0.01425***	
	(0.00257)		(0.00247)	
Race/Ethnicity: Other (1=Yes)	-0.00827		-0.00619	
	(0.00690)		(0.00908)	
Days Since Registering to Vote	-0.00001**		-0.00001**	
	(0.00000)		(0.00000)	
Missing Age (1=Yes)	-0.00648***		-0.00758***	
	(0.00249)		(0.00237)	
Missing Days Since Registering to Vote (1=Yes)	-0.01420***		-0.01629***	
	(0.00447)		(0.00449)	
Constant	0.03261***	0.01425***	0.03237***	0.01425***
	(0.00764)	(0.00127)	(0.00770)	(0.00127)
Observations	12,738	12,738	12,738	12,738
Weighted?	Yes	Yes	No	No
With Covariates?	Yes	No	Yes	No
Control Group Mean Turnout	0.0142	0.0142	0.0142	0.0142

Robust standard errors in parentheses. *** p<0.01, ** p<0.05, * p<0.1
The dependent variable is turnout in the 2014 general election, coded 1 if the subject voted and 0 otherwise.

C Sample Size Calculation

Following [1] (see p. 381), we assess whether the design is adequately powered (i.e., achieves 80% power) to detect a 1 percentage point effect given a control group mean turnout rate of 1.4%, a treatment group that is roughly 2.16 times the size of the control group (actual $n_T = 8704, n_C = 4034, n_T/n_C = 2.16$), and $\alpha = 0.05$.

Let p_C, p_T equal the proportion of subjects in control and treatment, respectively, who vote (i.e., for whom the binary dependent variable = 1); $\Delta = |p_T - p_C|$; n_C, n_T denote the target sample sizes for the control and treatment groups, respectively; α denote the probability of type I error; β denote the probability of type II error; z denote the critical value for a given α or β, and k denote the ratio n_T/n_C.

$$\begin{aligned}
n_C &= \frac{\left\{z_{1-\alpha/2} * \sqrt{\overline{p} * \overline{q} * (1 + \frac{1}{k})} + z_{1-\beta} * \sqrt{p_c * q_c + (\frac{p_T * q_T}{k})}\right\}^2}{\Delta^2} \\
q_C &= 1 - p_C \\
q_T &= 1 - p_T \\
\overline{p} &= \frac{p_c + k p_T}{1 + k} \\
\overline{q} &= 1 - \overline{p}
\end{aligned}$$

Substituting yields:

$$\begin{aligned}
n_C &= \frac{\left\{1.96 * \sqrt{0.0208 * 0.9792 * (1 + 1/2.16)} + 0.84 * \sqrt{0.014 * 0.986 + (\frac{0.024 * 0.976}{2.16})}\right\}^2}{0.01^2} \\
&\approx 2211 \\
n_T &= k * n_C = 2.16 * 2211 \\
&\approx 4776
\end{aligned}$$

Given the design, to achieve 80% power to detect an effect of 1 percentage point, we would need 2211 subjects in control and 4776 subjects in treatment. The actual sample sizes (4034 in control, 8704 in treatment) are about 1.8 times larger, which means that the design was adequately powered to detect a 1 percentage point effect.

References

[1] Rosner B. Fundamentals of Biostatistics. 7th ed. Brooks/Cole Cengage Learning; 2010.
